# The 4-Dimensional Plant: Effects of Wind-Induced Canopy Movement on Light Fluctuations and Photosynthesis

**DOI:** 10.3389/fpls.2016.01392

**Published:** 2016-09-21

**Authors:** Alexandra J. Burgess, Renata Retkute, Simon P. Preston, Oliver E. Jensen, Michael P. Pound, Tony P. Pridmore, Erik H. Murchie

**Affiliations:** ^1^Division of Plant and Crop Sciences, School of Biosciences, University of NottinghamLoughborough, UK; ^2^Crops for The Future, Semenyih Selangor Darul EhsanSemenyih, Malaysia; ^3^School of Life Sciences, The University of WarwickCoventry, UK; ^4^Centre for Plant Integrative Biology, School of Biosciences, University of NottinghamUK; ^5^School of Mathematical Sciences, University of NottinghamNottingham, UK; ^6^School of Mathematics, University of ManchesterManchester, UK; ^7^School of Computer Sciences, University of NottinghamNottingham, UK

**Keywords:** canopy, light, photosynthesis, wind, movement, mechanical canopy excitation, modelings

## Abstract

Physical perturbation of a plant canopy brought about by wind is a ubiquitous phenomenon and yet its biological importance has often been overlooked. This is partly due to the complexity of the issue at hand: wind-induced movement (or mechanical excitation) is a stochastic process which is difficult to measure and quantify; plant motion is dependent upon canopy architectural features which, until recently, were difficult to accurately represent and model in 3-dimensions; light patterning throughout a canopy is difficult to compute at high-resolutions, especially when confounded by other environmental variables. Recent studies have reinforced the expectation that canopy architecture is a strong determinant of productivity and yield; however, links between the architectural properties of the plant and its mechanical properties, particularly its response to wind, are relatively unknown. As a result, biologically relevant data relating canopy architecture, light- dynamics, and short-scale photosynthetic responses in the canopy setting are scarce. Here, we hypothesize that wind-induced movement will have large consequences for the photosynthetic productivity of our crops due to its influence on light patterning. To address this issue, in this study we combined high resolution 3D reconstructions of a plant canopy with a simple representation of canopy perturbation as a result of wind using solid body rotation in order to explore the potential effects on light patterning, interception, and photosynthetic productivity. We looked at two different scenarios: firstly a constant distortion where a rice canopy was subject to a permanent distortion throughout the whole day; and secondly, a dynamic distortion, where the canopy was distorted in incremental steps between two extremes at set time points in the day. We find that mechanical canopy excitation substantially alters light dynamics; light distribution and modeled canopy carbon gain. We then discuss methods required for accurate modeling of mechanical canopy excitation (here coined the 4-dimensional plant) and some associated biological and applied implications of such techniques. We hypothesize that biomechanical plant properties are a specific adaptation to achieve wind-induced photosynthetic enhancement and we outline how traits facilitating canopy excitation could be used as a route for improving crop yield.

## Introduction

Plant movement can be classed as autonomic (spontaneous) or occur as a biological response to stimuli. Here, movement most commonly refers to tropic, tactic, or nastic effects, which involve part of the plant, either an organ or organelle, responding to an external stimulus through processes of development. Such movements have been a popular focus for scientists because they involve key survival or adaptive mechanisms, including motion according to light, gravity, chemistry, or water. Charles Darwin was one of the first to document systematic experiments in this area in order to reveal underlying mechanisms (Darwin, 1880). Many years later and after enormous research effort we now understand this type of movement involves a highly sophisticated sensing and signaling system which allows the plant, over time, to grow, and position itself to optimize resource capture and competitive ability (Sussex and Kerk, 2001; Bhattacharya et al., 2010; Davies, 2013). However, the type of plant movement most immediately obvious to us is very different, and is that produced by physically perturbing the canopy, usually as a result of wind, here referred to as mechanical canopy excitation (Grace, 1977; de Langre, 2008). Despite its wide occurrence and a broad inter-disciplinary literature, there are many fundamental questions remaining concerning its effects on plant biology and especially photosynthesis. This class of movement can also occur in response to touch and a certain amount is known at the molecular level about signaling events involved (Knight et al., 1992; Chehab et al., 2012). Being a stochastic process, mechanical canopy excitation is difficult to quantify and measure and hard to link to fundamental plant processes.

The impact of wind on plants largely depends on speed, duration, and the extent to which wind can penetrate canopy layers. Sufficient wind speeds can affect plant development, form and function, resulting in reductions in leaf size, plant size (dwarfing), and damage to plant surfaces (Grace, 1977, 1988; Ennos, 1997; Smith and Ennos, 2003; de Langre, 2008; Onoda and Anten, 2011). High winds can also cause stem breakage and lodging (Berry et al., 2004), affect insect activity and population growth and the development and dispersal of pests and diseases within cropping systems (Aylor, 1990; Moser et al., 2009; Shaw, 2012). Wind alters heat and mass transfer, for example, by increasing leaf transpiration rate through reduction of boundary layer resistance, and the airflow regulates the microclimate of the vegetation (Grace, 1977, 1988; Brenner, 1996; de Langre, 2008). Moderate wind speeds can alter transpiration rates, indirectly affecting photosynthesis via changes in stomatal conductance and leaf temperature (Smith and Ennos, 2003) but this would be dependent upon the local environmental conditions. For example, in a hot environment, increases in transpiration and concurrent decreases in leaf temperature could be beneficial, assuming water is not limiting. However, under other less favorable conditions, the impact of moderate wind speeds on boundary layers alone may not affect leaf photosynthesis rates significantly or could negatively affect them (Grace, 1988).

A substantial impact of wind should arise through the altered light dynamics caused by movement of leaves (Roden, 2003; de Langre, 2008). Plant canopies are highly variable in terms of their light transmission characteristics, with leaf angle, clumping, density and leaf area index all playing a role in determining patterns of light extinction (Hirose, 2005). The spatial arrangement of plant material also creates a complex pattern of light components (direct, diffuse, and transmitted), typically resulting in progressively lowered light levels superimposed with high light patches or “light flecks.” There is also a predictable temporal effect caused by solar movement, which results in a spatial shifting of this pattern according to time of day. However, the true pattern of light within a canopy will depend upon these factors in combination with displacement brought about by wind. Early work predicted that alterations in leaf angle caused by wind can influence canopy photosynthesis (Caldwell, 1970). Roden and Pearcy (1993a,b) and Roden (2003) showed that fluttering leaves at the top of an Aspen tree canopy created an understory light environment that was more dynamic with a more even spatial distribution of light and enhanced photosynthesis in lower leaves that were adapted to utilization of rapid light flecks, plus reduced light interception at the top of the canopy. If mechanical canopy excitation is able to increase the probability of a photon penetrating into deeper canopy layers then it can be hypothesized that it can provide a method to increase photosynthesis. Such concepts have great significance for crop productivity but have not been explored in depth. Within the rest of this paper we will focus on small to moderate wind speeds (8–10 km h^−1^; 2–3 m^−2^ s^−1^), which are capable of facilitating mechanical canopy excitation, and thus potential photon penetration.

The “mosaic” of light patterns within a canopy can be predicted by ray tracing (e.g., Song et al., 2013) if one has a 3-dimensional computed reconstruction of the plant canopy. Recent developments in measuring 3-dimensional plant architecture at high resolutions are an essential tool in understanding canopy photosynthesis and crop improvement and can be used to predict dynamic responses of photosynthesis at the leaf level that scale up to the canopy (e.g., Falster and Westoby, 2003; Watanabe et al., 2005; Wang and Li, 2006; Sinoquet et al., 2007; Zheng et al., 2008; Pound et al., 2014; Burgess et al., 2015). However, these methods are currently limited to static plant descriptions and thus do not take into account mechanical perturbations to the canopy, such as those resulting from wind, despite it being a ubiquitous phenomenon.

In previous work we produced highly realistic 3-dimensional plant canopies of cereal plants and used these to predict light patterns and photosynthetic productivity (based on parameterisation with measured gas exchange data and en empirical model of photosynthesis) of architecturally contrasting lines (Burgess et al., 2015). High-resolution canopy descriptions have never been used to test the influence of mechanical canopy excitation on photosynthesis. Here we produce the first such simulation using a simple, solid body rotation as a first step toward providing the tools needed to predict the effect of wind on light patterning and photosynthesis. Within this study we aim to test the theory that mechanical canopy excitation may promote photosynthesis within crop canopies through altered light dynamics and that the effect is dependent on the amplitude and frequency of motion, thus could provide a route for future crop improvement.

If we assume that the number of possible 3-dimensional configurations is substantially increased by movement, then we hypothesize that it would result in an altered probability of direct photon penetration to lower layers (more potential routes for transmission). Previous work with leaf flutter in trees suggests that leaf motion would substantially increase the rate of light penetration (Roden and Pearcy, 1993a). We also hypothesize that a given surface area of leaf within the canopy is more likely to experience high light as a result of wind-induced perturbations. We discuss the impact these processes may have on the metabolism and physiology of leaves at the local scale, caused by an increase in the frequency of high light events.

## Materials and methods

### Growth of rice plants

We selected the commonly used rice variety IR64 for this study because it has a relatively upright leaf structure, which is likely to show typical responses to movement. This experiment took place during the summer of 2014 in a south—facing glasshouse at Sutton Bonington campus, University of Nottingham (52°49′59″N, 1°14′50″W) designed and built by CambridgeHOK (Brough, UK) for the growth of crop stands within a controlled environment. It consisted of a concrete tank 5 × 5 × 1.25 m positioned at ground level. The tank was filled entirely with a sandy loam soil, extracted from local fields and sieved through a fine mesh. Plants (cultivar IR64) were provided with adequate macro and micronutrients. Following soil analysis pre-experiment, additional elements supplied throughout the experiment were mostly N, P, K, and manganese. Plants were grown in nine plots, arranged in a 3 × 3 arrangement with 10 cm spacing between adjacent plants, a 1 × 1 m plot size and 10 cm spacing between adjacent plots. Watering took place via automated trickle tape application for 15 min twice a day. Supplementary sodium lighting was supplied (SON-T agro, Philips) at a position of approximately 3 m above ground level. Photoperiod in the glasshouse was regulated to 12 h using automated black out blinds. Temperature in the glasshouse was regulated to 30°C ± 3°C by automated venting and two gas-fired boilers. Humidity in the glasshouse was not regulated and typically varied between 60 and 70%.

### 3D reconstruction and modeling

**Figure 1:**
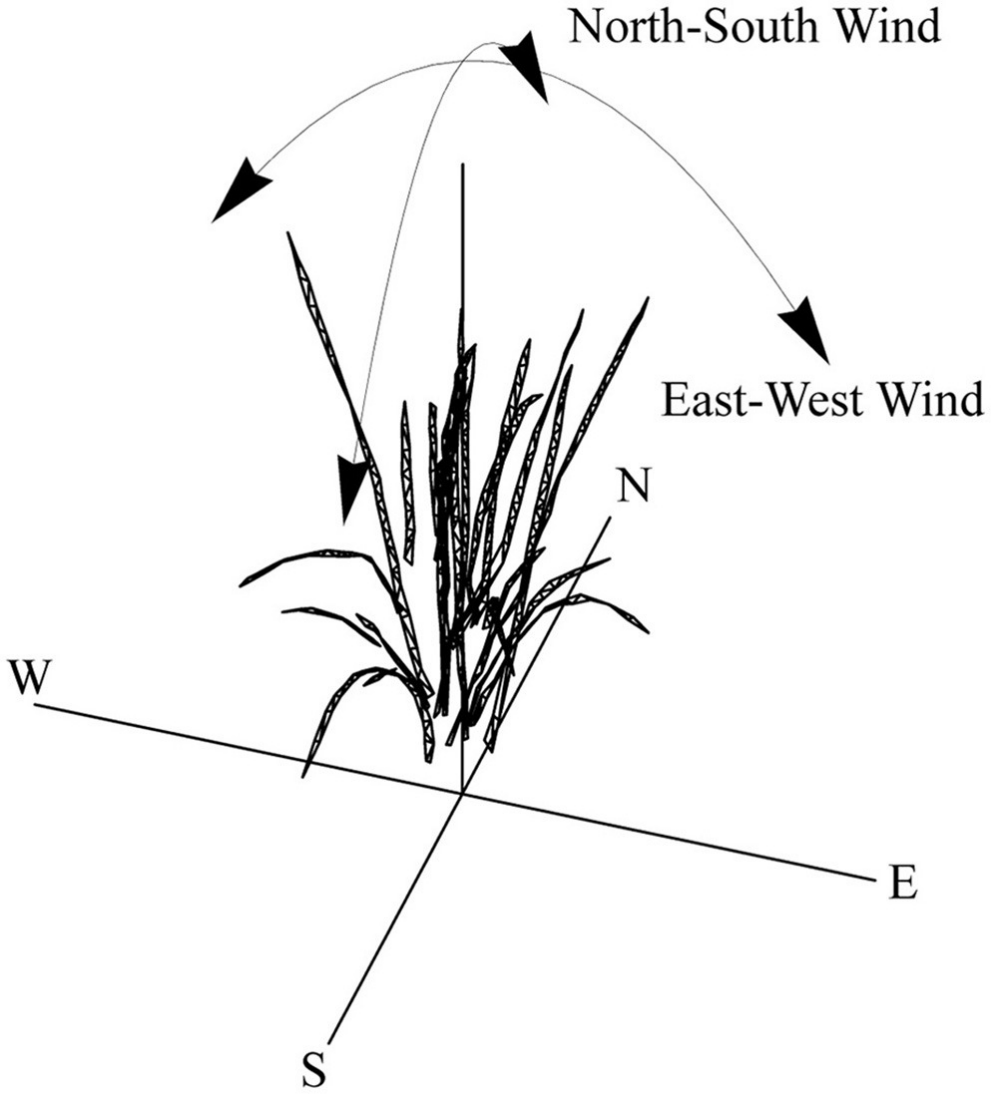
**Overview of solid body rotation distortion method**. Following distortion, 3 × 3 canopies were made.

A rice plant, grown as above, was subjected to 3D analysis (imaging and reconstruction) during a vegetative growth stage. The presence of panicles would have a significant effect but our aim was to focus on the effects of wind-induced movement on light patterning predominantly on leaf surfaces, but also to prevent errors with inaccurate reconstruction of panicles or potential inappropriate movement. This was made according to the protocol of Pound et al. (2014), which uses multiple RGB images as a basis for reconstruction. Topogun (2012) was then used to convert the rudimentary mesh into a cleaner mesh consisting of 600 triangles. The rice plant was duplicated and each of the nine duplicates were randomly rotated and assigned an identification number that referred to their layout on a 3 × 3 canopy grid (with set 10 cm spacing between plants). All duplicates were then distorted by solid body rotation, 1–10° about a set axis as shown in Figure 1 using Meshla (2014). This was used to simulate wind from a set direction. A forward ray-tracing algorithm, fastTracer (fastTracer version 3; PICB, Shanghai, China from Song et al., 2013), was used to calculate total light per unit leaf area throughout the canopies. Latitude was set at 14 (for the Philippines), atmospheric transmittance 0.5; light scattering 7.5%; light transmittance 7.5%; day 181 (30th June). To avoid interference from boundaries, we positioned the ray-tracing boundaries at centers of the outer plants. The software fires rays through a box with defined boundaries; when they exit one boundary (i.e., the side), they enter again from the opposite side.

Two different scenarios were modeled; firstly a constant distortion in which the canopy is subject to a constant wind causing a 6° distortion (equivalent to 7.6 cm displacement at the top of the canopy) throughout the whole day and; secondly a dynamic situation where the canopies were subject to a 0–10° (equivalent of 0–12.7 cm displacement) distortion (with 1° increments) at three time points throughout the day. For the constant displacement, the diurnal course of light intensities over a whole canopy was recorded in 1 min intervals and for the dynamic displacement, light intensities were recorded at 9:00, 12:00, and 15:00 h.

All modeling was executed in Mathematica (Wolfram).

Total canopy light interception per unit leaf area was calculated according to Equation 1.

(1) $$TL_{LA}=\frac{\sum_{i=1}^{n}S_{i}\int_{5.5}^{18.5}L_{i}(t)dt}{\sum_{i=1}^{n}S_{i}}$$

where *S*_*i*_ is the area of triangle *i.*

The response of photosynthesis to light irradiance, *L*, was calculated using a non-rectangular hyperbola given by Equation 2:

(2) $$\begin{array}{l} \begin{array}{l} \;F_{NRH}(L,\phi ,\theta ,P_{max},\alpha ) \end{array}\\ \begin{array}{l} =\frac{\phi \;L+(1+\alpha )P_{max}-\sqrt{{\left(\phi L+(1+\alpha )P_{max}\right)}^{2}-4\theta \phi L(1+\alpha )P_{max}}\;}{2\theta }-\alpha P_{max} \end{array} \end{array}$$

The non-rectangular hyperbola is defined by four parameters: the quantum use efficiency, ϕ; the convexity, θ; the maximum photosynthetic capacity, *P*_*max*_ and; the rate of dark respiration, *Rd*. We assumed that the rate of dark respiration is proportional to the maximum photosynthetic capacity, according to the relationship *Rd* = α *P*_*max*_ (Givnish, 1988; Niinemets and Tenhunen, 1997; Retkute et al., 2015). To maintain realism for the field we used the *P*_*max*_ photosynthetic parameter from IR72 canopies (Murchie et al., 2002, 32 for top layer, 21 middle layer, and five bottom layer). IR72 and IR64 lines give highly similar photosynthetic values (data not shown). ϕ was fixed at 0.052, θ at 0.845, and α to 0.1 for all canopy layers as these values represent the maximal value possible based on an uninhibited state (Leverenz, 1994; Werner et al., 2001; Burgess et al., 2015). The light response curves for all three canopy layers are given in Supplementary Figure S1.

For the constant wind scenario, the carbon assimilation at triangle *i* was calculated by combining Equation 2 with the predicted Photosynthetic Photon Flux Density (PPFD) at triangle *i* for each minute. Daily carbon assimilation, *P*_*i*_ (Equation 3), was then calculated by integrating the rate of photosynthetic carbon uptake over the day and multiplying by the area of the triangle, *S*_*i*_.

(3) $$P_{i}=S_{i}\int_{5}^{22}F_{NRH}\left(L_{i}(t),\phi ,\theta ,P_{max},\alpha \right)dt$$

As each canopy was divided into three layers, each triangle from the digital plant reconstruction was assigned to a particular layer, *m*, according to the triangle center (i.e., with triangle center between upper and lower limit of a layer depth). Carbon gain per unit leaf area was calculated as daily carbon assimilation over a whole canopy divided by the total surface area of the canopy according to Equation 4.

(4) $$C=\frac{\sum_{i=1}^{n}P_{i}}{\sum_{i=1}^{n}S_{i}}.$$

For the dynamic wind scenario, carbon gain was calculated from PPFD values using the light response curves as described by the non-rectangular hyperbola (Equation 2).

**Figure 2:**
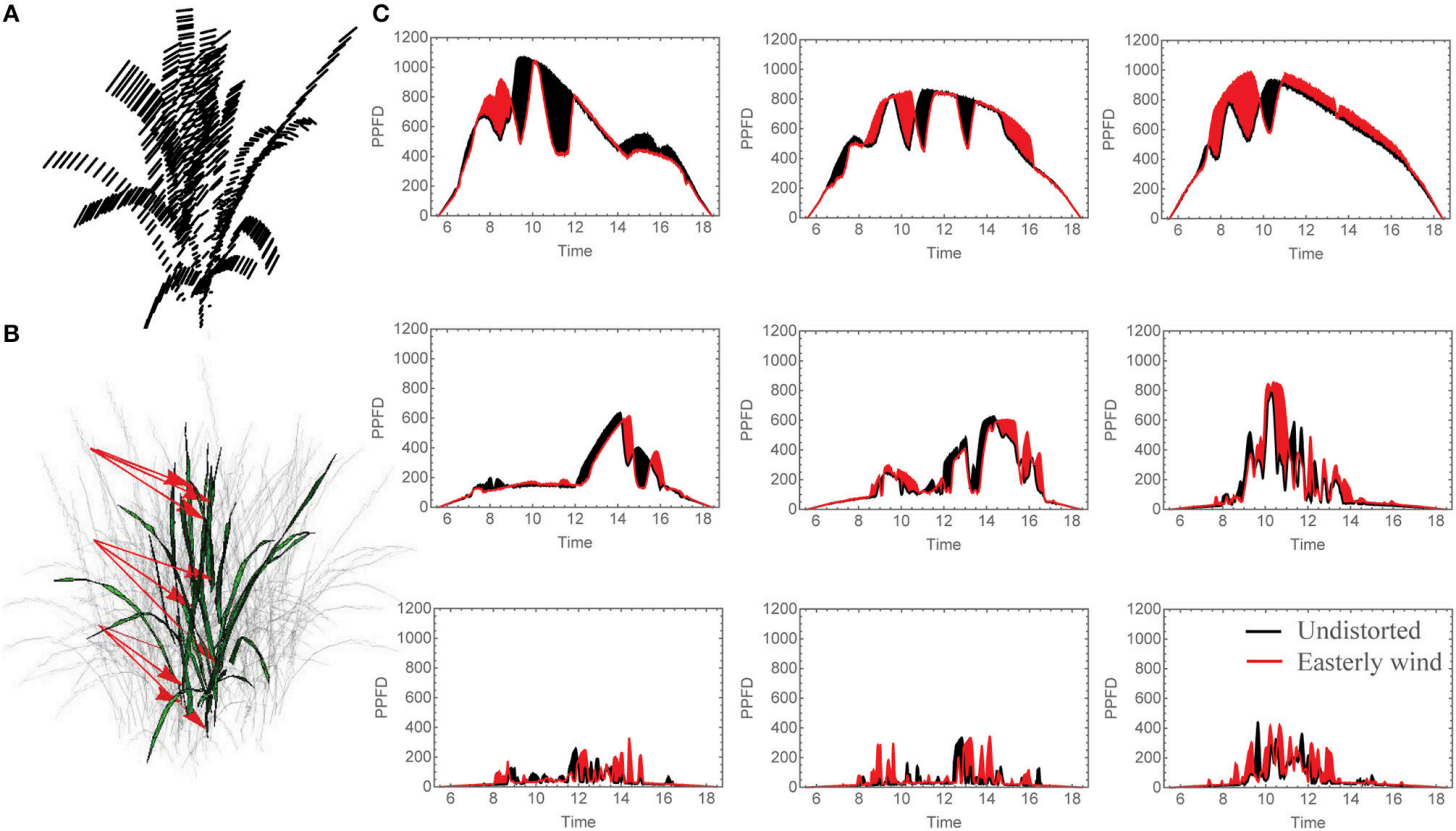
**Changing Light Patterns due to simulated Easterly wind. (A)** Schematic of plant distortion indicating center of each triangle before and after simulated mechanical canopy excitation, **(B)** leaf locations analyzed for light patterns given in **(C)**, the selected light patterns over the whole day where black shaded regions indicate a period of higher light intensity in the undistorted orientation (no wind) and red indicates a period of higher light intensity for the distortion corresponding to an easterly wind. N.B. The three grid strata in **(C)**. Correspond to the canopy strata as indicated by the arrows in **(B)**.

**Figure 3:**
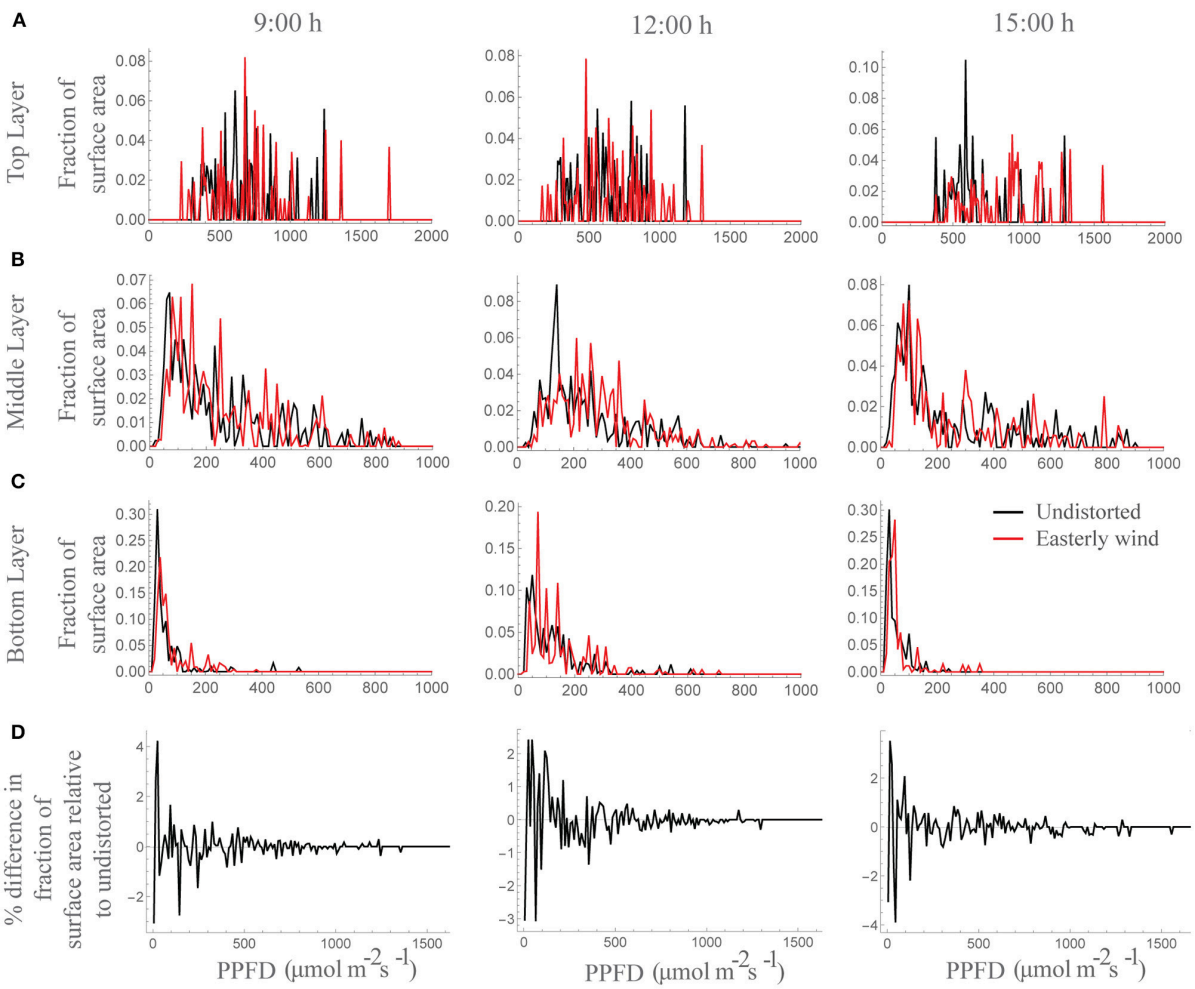
**Frequency of PPFD values according to the fraction of surface area received at by a whole plant within a canopy at 9:00, 12:00, and 15:00 h. (A)** Top layer, **(B)** middle layer, and **(C)** bottom layer where black is the undistorted canopy and red is the distortion equivalent to an easterly wind. **(D)** Percentage difference in the fraction of the total surface area receiving each PPFD value relative to the undistorted state; i.e., positive values indicate a higher surface area of the easterly wind distorted canopy receiving that set level of PPFD and negative values indicate a higher surface area of the undistorted canopy.

**Figure 4:**
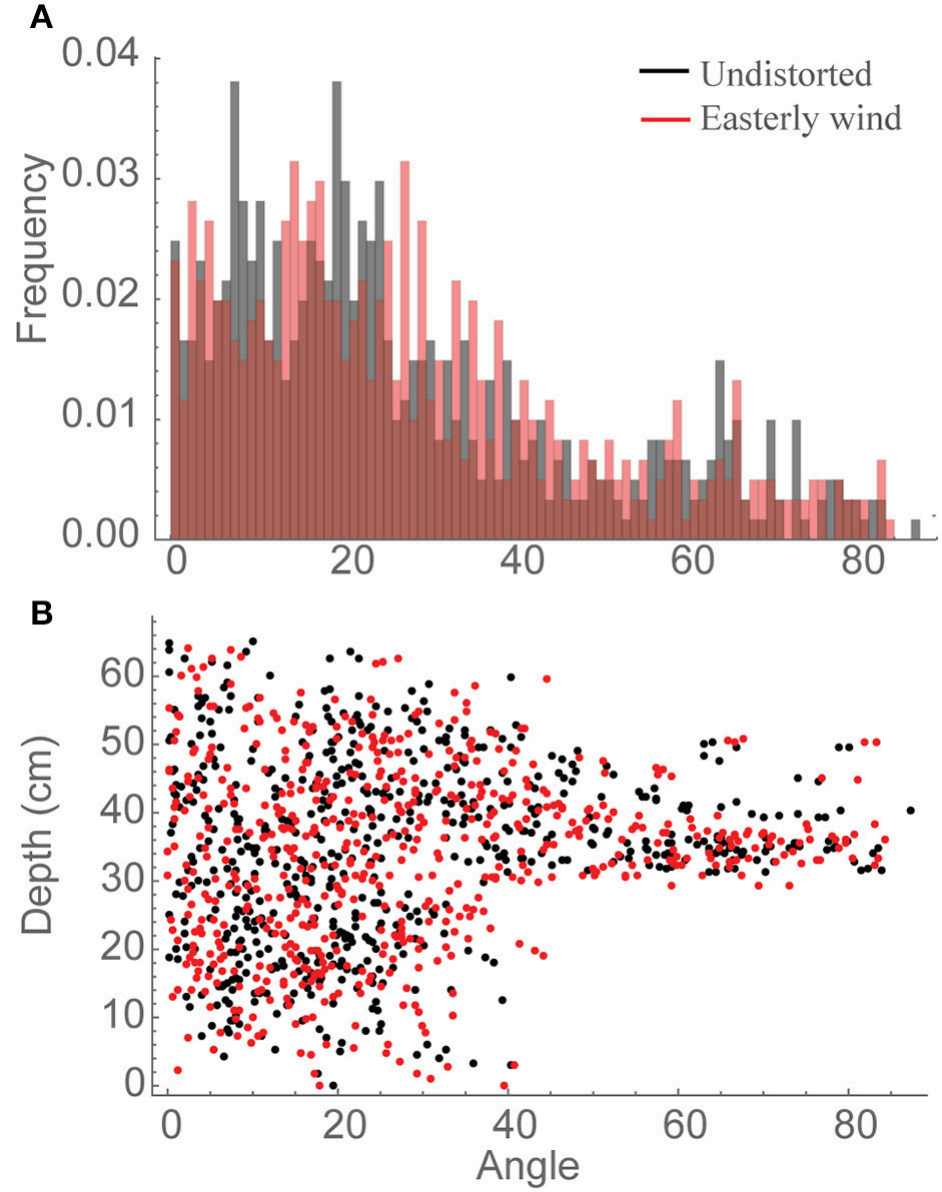
**Angle distributions relative to vertical, whereby 0 indicates a vertically inclined leaf section and 90 represents horizontally inclined leaf sections**. Data are shown for all canopy locations in the central plant of an undistorted canopy (black) vs. a canopy subject to an easterly wind (red) where **(A)** frequency of different leaf orientations and **(B)** distribution of individual leaf sections with depth through the canopy from the top.

**Figure 5:**
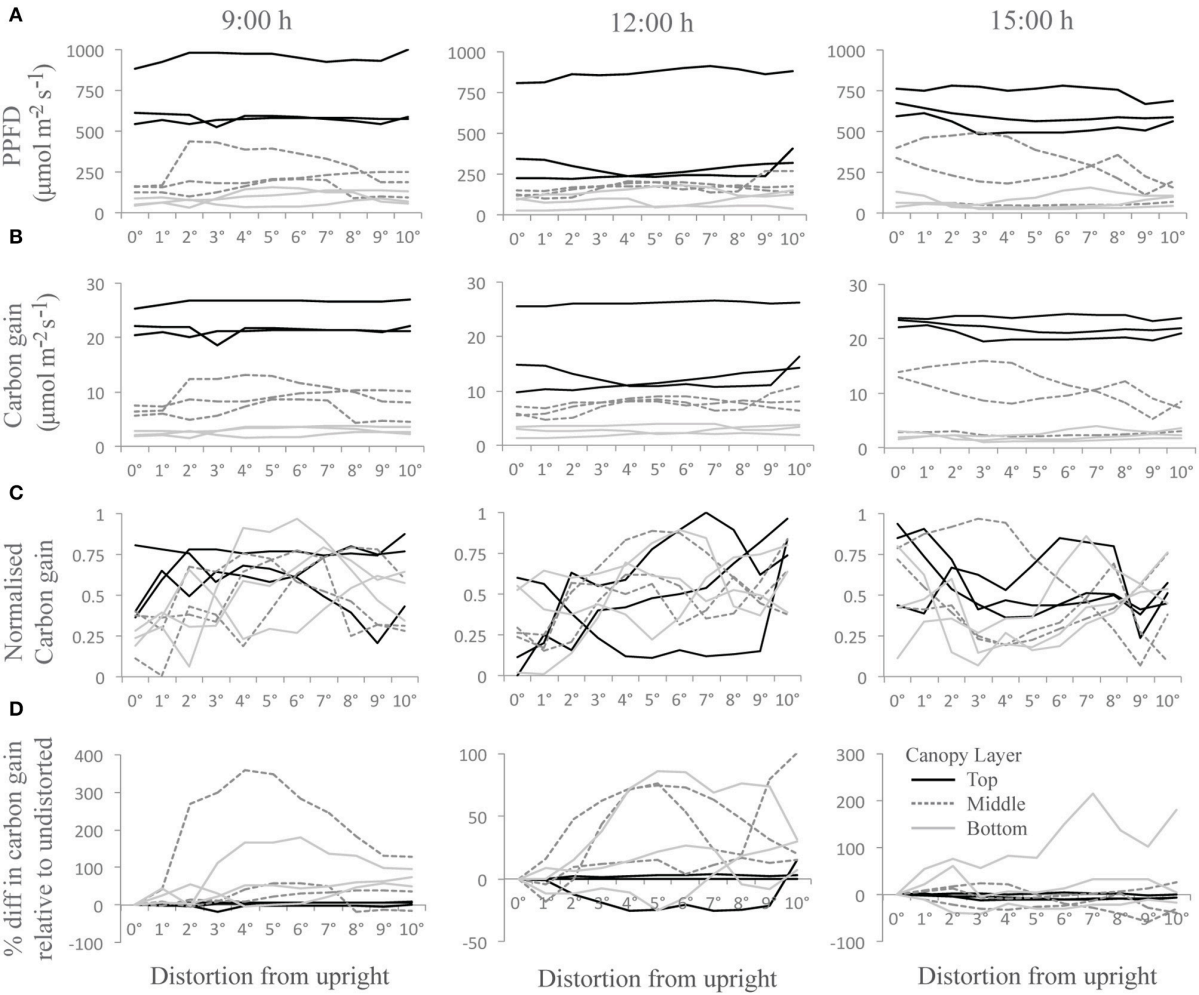
**Changes as a result of dynamic movement at three time points throughout the day and nine canopy locations (A) PPFD, (B) Carbon gain, (C) normalized carbon gain and (D) percentage difference in carbon gain relative to the undistorted state; where each line represents the average of five measurements from adjacent triangles on the same section of leaf; from the top (black line), the middle (dark gray and dashed line), and the bottom of the canopy (light gray line)**. Normalised carbon gain was calculated as carbon gain per individual location and averaged across the 5 locations in close proximity on the same leaf (i.e., the 5 locations represented by a single line). Data is presented for 3 different locations (i.e., 3 different leaves) per canopy layer. Values approaching 0 indicate the least favorable orientation in terms of carbon gain and 1 indicates the most favorable.

Data presented in Figures 2–**5** uses a simulated easterly wind, the predominant wind direction in the Philippines.

## Results

An overview of the distortion method is given in Figure 1. The before and after positions of each location in the rice canopy subject to a 6° distortion by an easterly wind is given in Figure 2A. We used ray tracing (FastTracer3; Song et al., 2013) and an empirical model of photosynthesis parameterised by measurements of photosynthetic gas exchange (see Materials and Methods) to determine the differences in light dynamics and overall carbon gain of the canopy.

The number of possible canopy configurations is extremely large. To introduce as much sensitivity as possible we have modeled two different scenarios; a constant wind inducing canopy displacement over the whole day and a dynamic wind, inducing varying degrees of displacement at three set time points during the day.

### Constant displacement

Under all wind directions tested, the moderate displacement (6°; equivalent to 7.6 cm displacement at the top of the canopy) resulted in changes in the light patterning at different canopy depths. Figure 2C shows the light signatures from nine different locations (those denoted by arrows in Figure 2B) in an undistorted (upright) canopy relative to a canopy subject to an easterly wind, where black shading indicates periods where there is a greater light intensity received in the undistorted canopy and red shading indicates a period of greater light intensity received by the distorted canopy. To explore this further, the frequency of PPFD values according to the fraction of surface area received by the central plant in the 3 × 3 canopy is shown for 9:00 h, 12:00 h and 15:00 h (Figure 3). For all three time points and canopy layers shown, there is a shift in the distribution toward a higher amount of intercepted light for the canopy subject to a constant moderate easterly wind relative to the undistorted canopy. This can also be seen over the course of the whole day as an increased total canopy light interception and translates into increases in total canopy carbon gain (Table 1; see Materials and Methods). The results obtained are dependent upon latitude, time of year, exact wind direction, and the exact configuration achieved for a given wind speed. The percentage difference in canopy surface area receiving a set PPFD relative to the undistorted canopy at each time point was also calculated (Figure 3D) where positive values indicate a higher surface area of the easterly wind distorted canopy receiving that set level of PPFD and negative values indicate a higher surface area of the undistorted canopy. This indicates that a greater surface area of the distorted canopy is under higher irradiance values relative to the undistorted canopy. These results support the hypothesis above that a mechanically excited canopy alters the light distribution patterns, here by altering the probability of a photon penetrating the canopy and being absorbed by leaves lower in the canopy.

**Table 1:** **Daily carbon gain per unit leaf area and total canopy light interception for each of the simulated wind directions**.

**Wind direction**	**Daily carbon gain per unit leaf area (mol m^−2^ d^−1^)**	**Total canopy light Interception per unit leaf area (mol m^−2^ d^−1^)**
None	0.262	7.548
West	0.279	8.014
East	0.307	9.325
South	0.301	8.905
North	0.285	8.297

Whilst it is not clear what is causing the increased interception of light in the distorted canopies we can speculate that it will be due to the more favorable leaf orientations. To assess whether this is the case, leaf angle distributions (Figure 4) were calculated relative to vertical (i.e., 0° represents an upright leaf and 90° a horizontally orientated leaf). These distributions do indicate a tendency toward more horizontal leaf orientations in the canopy subject to an easterly wind relative to an undistorted canopy (Figure 4A), which could be beneficial in this canopy where LAI is approximately 4. Distributions can also be calculated as a function of depth (Figure 4B), which, though difficult to interpret, indicate the possibility of more vertical leaves at the top of the canopy and more horizontal leaves at the bottom of the canopy in the easterly wind distortion. This trait (i.e., erect leaves at the top of the canopy and horizontal leaves at the bottom) represents the theoretical optimal structure for enhancing light interception and canopy photosynthesis as it provides a structure in which incident radiation can be uniformly distributed throughout all canopy layers (e.g., Duncan, 1971; Nobel et al., 1993). This indicates that the increase in light interception and carbon gain witnessed under a constant distortion could be a result of more favorable orientations of leaf material for the interception of light although the next section will be able to determine this further.

### Dynamic displacement

The “constant” displacement used above is useful but only partly representative of natural conditions. It represents states at either side of a continuum of movement. Significant computing power would be needed to calculate the effect of continually shifting between intermediate states over the course of the day since the number of configurations is so large. In our experiment we can anticipate that the actual values for carbon gain and total canopy absorption would be within this range of values. However, to assess how dynamic movements could affect the light environment and photosynthetic productivity, we have distorted the canopy by 1° increments from 0 to 10° (equivalent to 0–12.7 cm displacement at the top of the canopy) at three set time points throughout the day. We assume that the canopy will move through these positions within seconds consistent with the measured frequency under a light wind (data not shown) and too fast for a change solar angle to have a measurable effect. Supplementary Movie S1 shows a short animation of the modeled dynamic mechanical excitation with a single, central plan in bold and colored according to the maximum PPFD ranges each leaf portion is subject to. To indicate how degree increments in distortion can affect the light environment throughout the canopies three leaf locations per layer (located near those denoted in arrows in Figure 2B) were selected and the average of five PPFD values of adjacent triangles (taken from ray tracing data) was calculated (Figure 5A). Each PPFD value was translated into a carbon gain value using the light response curve as described by the non-rectangular hyperbola (Figure 5B; see Materials and Methods). These results show similar patterns as an increase in PPFD translates into an increase in carbon gain, although the magnitude of change will depend upon the region of the light response curve in which the point falls (i.e., a small change in PPFD can lead to a large change in carbon gain during the initial, linear phase of a light curve but will result in small differences in the saturating portion of the light curve; see Supplementary Figure S1 for the light response curves used in this study).

To see whether any orientation provides more favorable conditions, the normalized value of carbon gain was calculated (Figure 5C). This was calculated per individual triangle; each line represents the average of five triangles in close proximity on the same leaf. Values approaching 0 indicate the least favorable orientation in terms of carbon gain and 1 indicates the most favorable (N.B. each line represents the average of five measurements so does not reach the maximal limits). Whilst there is a lot of variation within the different canopy locations, there is a trend for an increase in the normalized values at 9:00 and 12:00 h but a decrease at 15:00 h from 0 to 10°. The full impact on carbon gain in different regions of the canopy can be seen by calculating the percentage difference in carbon gain of each distortion relative to the undistorted state (i.e., at 0°; Figure 5D). Negative values indicate where the distortion achieves a lower carbon gain relative to the undistorted state and can be seen most easily at 12:00 h at a position in the top of the canopy. The areas under the lines indicate the extra carbon gained as a result of shifting between 0 and 10°. The largest differences in carbon gain are found in leaf portions in the center and bottom of the canopy (as indicated by the dark gray and light gray lines, respectively), with an increase of 350% for a section of leaf from the middle of the canopy at 9:00 h. These results indicate that, using the wind-induced canopy configurations shown here, movement would result in the greatest alterations at the middle and the bottom of the canopy consistent with the hypothesis that foliage movement will increase the probability of photon penetration through to lower canopy layers. These simulations also indicate the importance of solar angle in determining how beneficial movement will be, with results from 15:00 h showing less beneficial effects relative to the other time points. Thus both the direction of movement and the solar angle will be important in determining the exact impact of wind on plant productivity.

## Discussion

From these findings we can deduce an impact on canopy light distribution resulting from a moderate wind speed. Our first hypothesis for the effect of mechanical canopy excitation was the increased probability of light penetration through canopy layers in a given time period, with more frequent movements leading to a higher probability of penetration. In the case of the data shown here for the constant scenario, the undistorted configuration (i.e., a canopy in its resting state) had substantially lower total canopy light interception and canopy carbon gain than the distorted configurations. However, this was not necessarily expected and whether a configuration is favorable or not is likely to result from the original characteristics of the undistorted canopy. A canopy may constantly shift between less and more favorable configurations the likelihood of each being, in turn, dependent on solar angle and wind characteristics. To test this hypothesis further we used a simplified simulation of a dynamic canopy which also showed that summing the incremental degrees of movement from the upright position can indeed lead to increased light interception, with the most profound positive effects being seen in the middle and bottom layers of the canopy (Figure 5D). Whilst we found no single leaf orientation to be the most favorable for all areas of the canopy explored, in our case the distortions were, in the most part, beneficial. This simulation also agrees with our second hypothesis: that a given surface area of leaf within the canopy is more likely to experience, on average, higher light levels than if the canopy were not able to move. However, for a full picture, the frequency of movement between states will ultimately determine productivity.

The effects of wind are likely to be very different for different canopy types. A planophile canopy with flatter leaves and a high extinction coefficient (Hirose, 2005) should offer less opportunity for light penetration since distortion is unlikely to alter leaf angle substantially. An erectophile canopy with a low extinction coefficient, like rice, is different: here upright leaves will absorb more or less light dependent on their angle, and hence position relative to the sun and importantly are more likely to influence penetration to lower layers. The canopy selected here has upright leaves at the top, progressing to more horizontal at the base, hence we were more likely to see the impact of displacement over time on the lower leaves. The next factor to consider is the biomechanical properties of the leaves and stems e.g., “stiffness,” which determines the frequency and amplitude of movement.

We have so far considered the probability of penetration of light into the canopy over time. For a given leaf surface, mechanical canopy excitation is likely to alter the dynamics of light patterning in terms of frequency, duration, and amplitude of high light periods. A high light event becomes more likely as the canopy starts to move but the average duration may be lower. The effect (and possibly biological function) of movement, especially in upper layers, then becomes one of light scattering and distribution. A simple analogy is that of a dance-room “mirrorball” spinning at fast or slow speeds. The faster it spins the more likely any area will receive a brief period of high light. In the next section we consider how such fluctuations will affect photosynthesis at the leaf level and how this can be manipulated in order to improve crop productivity.

### Mechanical canopy excitation: a means to manipulate photosynthesis?

Photosynthesis is considered to be a significant trait for crop improvement (Zhu et al., 2010). Since it is a dynamic process, any changes in local frequency or amplitude of illumination are linked directly to metabolic and physiological processes (such as stomatal opening, Rubisco activation, and photoprotection), governing the efficiency with which photosynthesis tracks the light in the plant canopy. The potential impact of mechanical canopy excitation on photon penetration leads to the exciting possibility that traits associated with movement, or response to movement, can be manipulated as a means to improve productivity of our cropping systems. This could be targeted at two core areas; firstly at metabolic features of the crop plants that enable them to exploit the short-term peaks in light intensity and respond rapidly to a change in light levels or; secondly at mechanical or architectural features of the crop stand that increase the probability of these high light events.

It has been shown both empirically (Hubbart et al., 2012; Lawson and Blatt, 2014) and theoretically (Zhu et al., 2004) that the speed of photosynthetic induction to and recovery from high light determines photosynthesis and water use efficiency. Genetic variation in photosynthetic induction rates means that the overall effect of this process will differ between species and lines, and be influenced by the frequency and intensity of high light events. In the understorey, such light flecks can make up 90% of available light (Pearcy, 1990). Higher frequencies should reduce the wastage caused by slow induction and relaxation (Murchie et al., 2009).

Our data suggests that mechanical canopy excitation could also provide a means to substantially alter and even improve light penetration in crops relative to canopy structures that do not facilitate movement. The distribution of light in a canopy is a determinant of photosynthesis and productivity (Zhu et al., 2010; Song et al., 2013). Past studies indicate that the ideal canopy system is one with a high leaf area index but efficient light penetration in order to avoid saturation of photosynthesis at the top and avoid severe light limitation at the base. This established principle has led to the suggestion that to improve crop yield, leaf chlorophyll concentration at the top of the canopy should be lowered to aid light penetration whilst efficient light harvesting should be maintained at the bottom (Ort et al., 2015). However, improved light distributions within the canopy could also be achieved by manipulating the biomechanical properties of the crop. This is consistent with the fluttering leaves example in Aspen trees of Roden and Pearcy (1993a,b) and Roden (2003). The implication is that the more rapid the leaf movement the greater the probability of light penetration. This is also consistent with our work, and suggests that we should select for leaf properties in crops that permit small but rapid movements in light winds, similar to leaf flutter, although the exact traits will depend upon the canopy type (e.g., see planophile vs. erectophile canopies above) although could include traits such as sheath or petiole flexibility, stem strength plus altered leaf blade length, and width. It would be anticipated that stiffer stems and leaves would lead to lower frequencies and amplitudes of movement in light winds. Substantial variation for biomechanical properties exists in cereals and can result from such properties as stem wall thickness and non-structural carbohydrate content (Kashiwagi et al., 2008). Within the Aspen studies, the lower leaves were typically acclimated to high light with fast photosynthetic induction times, limited largely by Rubisco activation rather than stomata. This last point is important since the frequency of switching between high and low light will determine the “drag” effect of photosynthetic induction: a higher frequency can lead to a higher integrated photosynthetic rate. Such improvements could be incorporated into crop plants by using existing variation in biomechanical properties (Berry et al., 2004). Under moderate wind speeds this can be achieved without a risk to stem failure: our suggested changes need not involve a compromise to stem strength since they could be achieved by changes to upper part of the canopy alone.

The evolutionary or ecological significance of divergent groups of plants could be explored by studying differences in their modes of movement. For example, tree species have relatively solid trunks, thus movement is largely limited to the leaves. In contrast, cereal or other crop canopies could rely on wind to facilitate movement of the whole canopy although this should not be at the risk of increased stem failure. Crops have been cultivated under field conditions and so the selection process may already have incorporated wind as a factor. However, it has been argued for a long time that photosynthesis per unit leaf area has not undergone genetic improvement as a result of “surrogacy” by other physiological and morphological improvements (Reynolds et al., 2000). There is no reason to assume that the same does not apply to wind and mechanical motion with respect to photosynthesis. By studying model species such as rice and wheat with complex canopies and genetically altered canopy mechanical properties as well as wild relatives it will be possible to empirically test such theories further. However, for precise predictions of the effect of wind-induced movement on canopy photosynthesis we need to build more realistic distortions of the plant that are informed both by mechanical models and observations of real canopy motions described below.

### The technology required for simulating the 4-dimensional canopy

Here, we have used a simple distortion based on solid body rotation as a means to predict the effect of mechanical canopy excitation on the resulting light environment. However, this type of movement is not realistic and does not reflect the unique and complex perturbations that plant material is subject to. Whilst the technology to reproduce this form of movement for these purposes is not currently available, we can predict what would be required. In order for mechanical canopy excitation to be incorporated into studies of canopy photosynthesis it is necessary to have a model that can accurately mimic a wide range of movements. Whilst movement may at first appear simple, in reality wind-induced displacement is highly complex, and involves interactions between the individual mechanical properties of organs and plant, the characteristics of the wind and the physical proximity of other plants. For example: leaves may bend in different ways (partly dependent on growth angle); leaves are displaced at different rates in relation to each other; wind speed and direction are very complex and can change rapidly over short time scales; the characteristic features of canopy architecture can change throughout growth and development; solar angle changes throughout the day and year meaning that the light patterns will alter even if wind speed and direction remains the same.

For accurate modeling of movement, mechanical properties of individual organs and the canopy as a whole must be incorporated (de Langre, 2008). Firstly, one needs to mimic the distortion of leaves that are thicker and stiffer at their base. A simple representation of a leaf is as a tapered inextensible elastic rod that resists bending and that is anchored at its base. The rod can have an intrinsic curvature and is also bent by gravity. In the presence of wind, the rod will distort under drag forces from the air. These are likely to fluctuate because of turbulence. Furthermore, interactions between fluid and structural forces are likely to induce instabilities of the rod (via a form of aerodynamic flutter), which can typically be described using a small number of characteristic modes of oscillation of the rod. So the distortion of leaves in a real airflow will (a) be greater toward the more flexible tips of leaves, (b) will typically be unsteady, and (c) will be complicated by leaf-leaf interactions. These mechanical properties will differ based upon the crop used for study and its specific architecture. For example, the majority of rice leaves will be anchored toward the center and base of the plant whereas wheat leaves will be anchored to a specific point on a stem- an organ, which will also have its own mode of movement, separate to those of the leaves.

Before accurate mathematical models can be produced, plant movement must first be captured. Methods and tools are required that are capable of characterizing the motion of individual plant components in response to wind. This can be achieved through the development and application of appropriate visual tracking technologies. Visual tracking methods seek to maintain a description of the identity and movement of one of more target objects through a time-ordered image sequence (Yang et al., 2011). Classic applications of visual tracking involve tracking hands and faces for human-computer interaction, and whole bodies for security and surveillance. Tracking can occur in any number of dimensions; one method might track the apparent movement of a leaf across the (2D) image, another the motion of a (3D) surface patch describing that leaf. Tracking methods can consider the whole object of interest, or a number of distinct parts (e.g., limbs, torsos) separately, combining results to give a final description of the target's motion.

Characterisation of individual and plant canopies' response to wind requires the position and orientation of surface patches extracted from multiple views to be tracked in 3-dimensional space. Hypothesised patch properties must be verified by reference to the available image(s) (as used in Pound et al., 2014). The similar appearance of plant components make this a challenging task, but one that is within reach given current computer vision methods. To provide a full description of the effects of wind, differences in leaf shape and stalk shape must be recovered by comparison of the 3-dimensional plant descriptions obtained, and tracked, over time. Again, this is challenging, but within reach of current methods. By careful consideration of individual and sets of 2-dimensional images of wind-excited plants over time we can aim to provide the rich descriptions of canopy motion that are needed to understand its effect on photosynthesis. Such methods, when combined with light (or other environmental) modeling could be used to build a true “four-dimensional plant”.

The concept of a “four-dimensional plant model” may not be as far into the future as we think. Whilst both the mathematical and computational methods are attainable with current technology, biologically relevant data are also integral. For example, details on wind speed and direction will be critical for accurate modeling, as well as the growth conditions and management practices of the crops under investigation (e.g., Sterling et al., 2003). Whilst knowledge of the physical conditions of the canopies is required, the biological, and biochemical properties of the crops are also important. Whilst this study has mainly focused on the altered light dynamics brought about by wind-induced movement, there are other environmental variables that would also be influenced. For example, turbulent airflow throughout canopy structure will have implications in terms of altered CO_2_ and O_2_ flux to leaves. Canopy CO_2_ depression may be mitigated and similarly, transpiration rate and vapor pressure deficit in different canopy regions could also be altered.

The extent to which productivity will be affected will depend upon the local environmental conditions. Here we looked at latitude 14 (corresponding to the Philippines) but as solar angle and intensity is determined by latitude and the time of year, the location and season under study could influence the final productivity, and different modes of movement may be more suitable for a set location. For example an upright canopy (static) is particularly efficacious at lower altitudes due to the enhanced light penetration. However, we would predict that movement will become more beneficial for upright canopies as we move to higher latitudes with low solar elevation. Furthermore, *fastTracer3* (i.e., ray tracing; Song et al., 2013) assumes a constantly sunny day thus the values presented in this study are likely to represent the extremes for direct light. However, in reality cloud cover will alter the intensity and spectral composition of light reaching the top of a canopy, for example through an increase in the proportion of diffused light. In some environments the development of cloud cover throughout the day is predictable. Therefore, models informed by both weather data and the biological response of acclimation state; to the effect of altered light patterning and; to changes in airflow on photosynthetic productivity, will be essential for fully assessing the influence of wind at the whole canopy level.

There are a number of applications for high-resolution models of mechanical canopy excitation that mean that such 4-dimensional modeling techniques will be the foundation for detailed studies in a number of different areas. For example, such models can be used to better investigate how authentic, rapid oscillations between high- and low- light intensities affect the biochemical and physiological properties of plants, or how such changes alter the quality and quantity of different light components reaching leaves (e.g., direct, diffuse, and scattered light or the light spectrum at different canopy layers). They could also be used to explore the effect of different architectural types on movement and light patterning. Information could also be used to inform ideal plant types; it may be that leaves that have specific mechanical properties enabling greater movement during wind (especially light intensity wind) could enable greater light penetration under certain conditions and therefore such plants could better exploit the environment in which they are grown. This could further be adapted to better inform structural modeling to inform lodging models of the structural properties required to resist certain wind speeds or directions and thus engineer a more resilient plant. Applications may also extend to other areas such as making predictions on the effect of future climate change scenarios or extreme weather events on cropping systems (e.g., Willenbockel, 2012; Lizumi and Ramankutty, 2015) or predicting the effect of disease spread for wind- and water- dispersed pathogens during outbreaks (e.g., Legg, 1983; Shaw, 2012).

The modeling work carried out here indicates that relatively small perturbations within the canopy can substantially alter the light environment and associated productivity in a cereal canopy. However, for accurate predictions of the effect of wind-induced movement on canopy photosynthesis we need to build more realistic distortions of the plant that are informed both by mechanical models and observations of real canopy motions. Such approaches will be critical in accurately predicting whole canopy photosynthesis and exploring the effects of rapid changes in light intensity (i.e., those brought about by light flecks). This leads to the intriguing new possibility that manipulating the plant's mechanical movement properties in relation to wind, or the plant's response to rapid high light events, can be used as a means to optimize photosynthesis at the canopy level and therefore provide a route for crop improvement.

## Author contributions

AB performed most of the modeling work with the assistance of RR, OJ, and SP. provided information and knowledge on mechanical mathematical modeling; MP and TP provided information and knowledge on the hardware and technologies required for capturing plant movement and image analysis; AB and EM conceived and wrote the article with contributions of the other authors.

## Funding

AB is funded by Crops For the Future under project BioP1-006 and the School of Biosciences, U.Nottingham. EM, TP, OJ, SP receive funding from the Biotechnology and Biological Sciences Research Council (grant BB/JOO3999/1).

### Conflict of interest statement

The authors declare that the research was conducted in the absence of any commercial or financial relationships that could be construed as a potential conflict of interest.

## Supplementary material

Supplementary Movie S1 **Change in overall canopy light distribution due to an Easterly wind at 12:00 h between 0 and 10° distortion**.

Supplementary Figure S1 **Light response curves used to calculate canopy carbon gain for the top (black), middle (dark gray, dashed), and bottom (light gray) layers**. *P*_*max*_ values were taken from Murchie et al. (2002) and other parameters fixed as the maximal attainable value given an uninhibited state.
