# Supplementary figures and images for: The 4-Dimensional Plant: Effects of Wind-Induced Canopy Movement on Light Fluctuations and Photosynthesis
Source: Front Plant Sci. 2016 Sep 21;7:1392. doi: 10.3389/fpls.2016.01392 (PMC5030302; doi:10.3389/fpls.2016.01392)

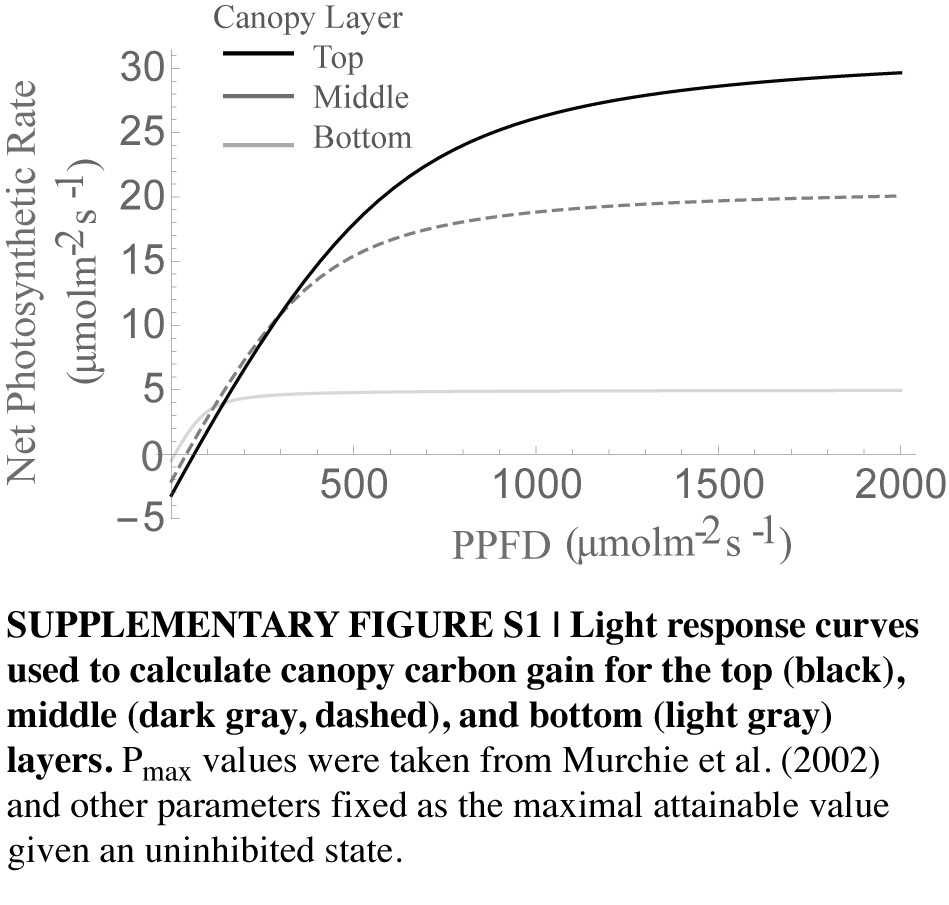

Supplement: Supplementary Figure S1 — Light response curves used to calculate canopy carbon gain for the top (black), middle (dark gray, dashed), and bottom (light gray) layers. Pmax values were taken from Murchie et al. (2002) and other parameters fixed as the maximal attainable value given an uninhibited state. [file Image1.tif]
